# Associations between respiratory symptoms, lung function and gastro-oesophageal reflux symptoms in a population-based birth cohort

**DOI:** 10.1186/1465-9921-7-142

**Published:** 2006-12-05

**Authors:** Robert J Hancox, Richie Poulton, D Robin Taylor, Justina M Greene, Christene R McLachlan, Jan O Cowan, Erin M Flannery, G Peter Herbison, Malcolm R Sears, Nicholas J Talley

**Affiliations:** 1 Dunedin Multidisciplinary Health and Development Research Unit, Dunedin School of Medicine, University of Otago, Dunedin, New Zealand; 2 Department of Medical and Surgical Sciences, Dunedin School of Medicine, University of Otago, Dunedin, New Zealand; 3 Firestone Institute for Respiratory Health, Department of Medicine, McMaster University, Hamilton, Ontario, Canada; 4 Department of Preventive and Social Medicine, Dunedin School of Medicine, University of Otago, Dunedin, New Zealand; 5 Mayo Clinic College of Medicine, Division of Gastroenterology and Hepatology, and Internal Medicine, Mayo Clinic, Rochester Foundation, Rochester, Minnesota, USA

## Abstract

**Background:**

Several studies have reported an association between asthma and gastro-oesophageal reflux, but it is unclear which condition develops first. The role of obesity in mediating this association is also unclear. We explored the associations between respiratory symptoms, lung function, and gastro-oesophageal reflux symptoms in a birth cohort of approximately 1000 individuals.

**Methods:**

Information on respiratory symptoms, asthma, atopy, lung function and airway responsiveness was obtained at multiple assessments from childhood to adulthood in an unselected birth cohort of 1037 individuals followed to age 26. Symptoms of gastro-oesophageal reflux and irritable bowel syndrome were recorded at age 26.

**Results:**

Heartburn and acid regurgitation symptoms that were at least "moderately bothersome" at age 26 were significantly associated with asthma (odds ratio = 3.2; 95% confidence interval = 1.6–6.4), wheeze (OR = 3.5; 95% CI = 1.7–7.2), and nocturnal cough (OR = 4.3; 95% CI = 2.1–8.7) independently of body mass index. In women reflux symptoms were also associated with airflow obstruction and a bronchodilator response to salbutamol. Persistent wheezing since childhood, persistence of asthma since teenage years, and airway hyperresponsiveness since age 11 were associated with a significantly increased risk of heartburn and acid regurgitation at age 26. There was no association between irritable bowel syndrome and respiratory symptoms.

**Conclusion:**

Reflux symptoms are associated with respiratory symptoms in young adults independently of body mass index. The mechanism of these associations remains unclear.

## Background

An association between symptoms of asthma and gastro-oesophageal reflux is now well-recognised, with a number of studies reporting a much higher prevalence of reflux symptoms in patients with asthma than in control subjects [1]. Objective measurements using endoscopy and oesophageal pH monitoring confirm a high prevalence of reflux in asthma [2,3].

The association between gastro-oesophageal reflux and asthma could have several explanations [4]. Reflux may precipitate asthma, either via a vagal reflex initiated by gastric fluid in the oesophagus, or by micro-aspiration of gastric contents into the trachea. Conversely, asthma may promote reflux due to the increased pressure swings in the thorax during respiration [5,6]. During 24 hour monitoring of oesophageal pH and asthma symptoms, reflux appeared to precipitate asthma symptoms and cough far more often than asthma precipitated reflux [7]. However, although episodes of gastro-oesophageal reflux might trigger wheezing in an individual who has asthma, this does not necessarily indicate an aetiological association between having reflux disease and the asthmatic phenotype. The association between asthma and reflux could also be mediated by obesity, which is a risk factor for both conditions [8-11]. Finally, reflux could give rise to asthma-like symptoms, such as nocturnal cough, but have no effect on lung function or airway responsiveness.

Population-based studies of asthma and reflux are rare. A postal questionnaire of British adults confirmed an association between reflux symptoms and symptoms suggestive of bronchial hyperresponsiveness and also found an association between respiratory symptoms and irritable bowel syndrome [12]. The European Community Respiratory Health Study found a strong association between symptoms of nocturnal reflux and asthma and respiratory symptoms in a random sample of young adults. This remained significant after adjustment for body mass index [13]. A recent follow-up study of the same participants 5 – 10 years later, found both obesity and nocturnal reflux symptoms to be independent risk factors for the onset of asthma [14].

We explored the relations between symptoms and objective evidence of asthma, reflux and obesity in the Dunedin Multidisciplinary Health and Development Study – a birth cohort of approximately 1000 individuals followed to age 26. We hypothesised that if asthma predisposes to gastro-oesophageal reflux, then long-standing persistent asthma would be associated with the highest risk of reflux symptoms. Conversely, if reflux precipitates asthma, adult reflux symptoms would be most strongly associated with adult-onset asthma.

## Methods

The Dunedin Multidisciplinary Health and Development Study is a cohort study of 1037 children (52% male) born April 1972 to March 1973 [15]. Follow-up assessments have been conducted at ages 3, 5, 7, 9, 11, 13, 15, 18, 21, and 26 years when 980 (96%) of 1019 living study members participated. The Otago Ethics Committee approved the study and written informed consent was obtained at each assessment.

At age 9, the accompanying adult was questioned about current and previous asthma, wheeze and cough [16]. This information was updated at subsequent assessments [17]. Current asthma was defined as diagnosed asthma with at least one episode of asthma or wheezing symptoms within the previous year. Current wheeze was defined current wheeze as episodes of wheezing in the last year, excluding those with only one or two episodes each lasting less than one hour. At age 26 Study members were asked if they had woken with coughing in the previous year when they did not have a cold. Current smoking was defined as smoking daily for at least one month during the past year. Cumulative lifetime smoking history was assessed as total "pack-years" smoked up to age 26 years where one pack-year is the equivalent of smoking one pack of 20 cigarettes every day for a year.

Height without shoes, and weight in light clothing, were measured to calculate Body Mass Index (BMI) in kg/m^2^. Spirometry to measure the forced expiratory volume in one second (FEV_1_) and forced vital capacity (FVC) was measured at ages 9, 11, 13, 15 and 21 years using a Godart water-sealed spirometer. At age 18 years spirometry was performed before and after nebulised salbutamol using a Morgan rolling seal spirometer. At age 26 years, spirometry was performed before and after salbutamol 200 μg inhaled from a metered dose inhaler via a valved spacer using a Sensormedics body plethysmograph. Airway responsiveness to methacholine was measured at ages 9, 11, 13, 15 and 21 years using a validated modified Chai protocol [18]. A provoking concentration of methacholine to induce a 20% fall in FEV_1 _(PC_20_) of 8 mg/mL or less indicated airway hyperresponsiveness. When methacholine challenge was not undertaken (at ages 18 and 26), or if a low FEV_1 _precluded testing at other ages for safety reasons, an increase in FEV_1 _of 10% or greater after inhaling salbutamol (bronchodilator response) was taken as also indicating airway hyperresponsiveness. Skin prick testing at age 21 included house dust mite (Dermatophagoides pteronyssinus), grass, cat, dog, horse, kapok, wool, Aspergillus fumigatus, alternaria, penicillium, and cladosporium [19]. A weal diameter 2 mm greater than saline control was considered positive and atopy was defined as a positive response to one or more allergens.

At age 26 Study members were asked questions from the Bowel Symptom Questionnaire [20]. These included whether they had had "heartburn (a burning pain or discomfort behind the breastbone rising up in the chest)" and if they had had "a bitter or sour tasting fluid that comes to your throat or mouth" (acid regurgitation). Each symptom was scored according to how bothersome it was: 0 = I have not been bothered by this symptom; 1 = A little bit bothersome; 2 = Moderately bothersome; 3 = Quite a bit bothersome; 4 = Extremely bothersome. For this analysis, reflux symptoms were included if they were at least moderately bothersome (score 2 and above).

Irritable bowel syndrome was defined using the Manning criteria [21]. This required abdominal pain or discomfort plus at least two of the following six symptoms: pain relief by defecation, looser stools at the onset of pain, more frequent stools at the onset of pain, abdominal distension, mucus per rectum and feeling of incomplete rectal evacuation. These criteria are highly specific for irritable bowel syndrome [22] and identify more cases than the Rome criteria [23].

### Statistical Analysis

Cross-sectional associations between asthma, wheeze, cough, bronchodilator responsiveness and the FEV_1_/FVC ratio and gastro-intestinal symptoms of heartburn, regurgitation and irritable bowel syndrome at age 26 were analysed by logistic or linear regression using the gastro-intestinal symptoms as the independent (predictor) variables. Heartburn and regurgitation symptoms were considered individually and in combination. All analyses controlled for sex and BMI. Analyses tested for interactions between sex and gastro-intestinal symptoms and, because of known sex differences in the association between body mass index and asthma in this cohort [9], analyses were repeated for each sex separately. We also tested for an interaction between reflux symptoms and atopy. Further analyses adjusted for smoking status.

Longitudinal associations between asthma, wheeze, and airway hyperresponsiveness at earlier ages and reflux symptoms at age 26 were examined by logistic regression. Asthma and wheeze were used as the independent (predictor) variables in these analyses and were categorised according to the age at which they were first reported and whether they were still present at age 26. Thus asthma was classified as "child-persistent" asthma if an asthma diagnosis was first reported at age 9 or 11 and still present at age 26, "teen-persistent" if first reported at age 13, 15 or 18 and still present at age 26, "adult-onset" if first reported at age 21 or 26 and "remittent" if asthma had been reported at an earlier assessment but not at age 26. The same classifications were made for wheezing. Associations between airway hyperresponsiveness at earlier assessments and reflux symptoms were analysed for each age using logistic regression. All analyses adjusted for sex. Further analyses of hyperresponsiveness adjusted for current asthma and symptoms of wheeze.

Pregnant women (n = 33) and Study members with symptoms meeting American Psychiatric Association criteria for eating disorders [24] (n = 25) at age 26 were excluded from all analyses. Analyses were performed using Stata version 9 (Stata corporation, College Station, TX).

## Results

### Cross-sectional associations at age 26

Heartburn and acid regurgitation symptoms that were at least "moderately bothersome" were reported by 12.5% and 6.0% respectively while 4.1% reported both (table 1). The frequencies of heartburn and acid regurgitation did not differ between men and women whereas irritable bowel syndrome was more common in women than men (20.3% and 13.6% respectively, p = 0.007, 95% CI for difference 1.9–11.6%). The prevalence of respiratory outcomes at age 26 years and the mean FEV_1_/FVC ratio in those with and without gastrointestinal symptoms are shown in table 1.

**Table 1:** Prevalence of respiratory outcomes in those with and without reflux symptoms and irritable bowel syndrome at age 26 years

	% prevalence	% with respiratory condition				Mean FEV_1_/FVC
		Asthma	Wheeze	Cough	BDR	
No Reflux symptoms*	85.8	17.2	35.0	12.4	6.9	81.7 %
Heartburn	12.5	28.3	48.7	23.9	14.2	80.9 %
Regurgitation	6.0	38.9	62.3	35.2	18.0	79.3 %
Heartburn and regurgitation	4.1	43.2	67.6	40.5	22.9	78.9 %
Irritable Bowel Syndrome	16.7	20.4	41.7	13.2	8.4	81.5 %

* excludes anyone reporting either bothersome heartburn or acid regurgitation. BDR (bronchodilator response) = 10% or greater increase in FEV_1 _following salbutamol.

Heartburn and acid regurgitation symptoms were significantly associated with a diagnosis of asthma, symptoms of wheeze and waking with a cough (table 2). These associations were similar in men and women and were independent of BMI. None of the respiratory outcomes was associated with irritable bowel syndrome.

**Table 2:** Association of asthma diagnosis and respiratory symptoms with reflux symptoms and irritable bowel syndrome at age 26 years

		Heartburn		Regurgitation		Heartburn and regurgitation		Irritable Bowel Syndrome	
	n	OR (95% CI)	p	OR (95% CI)	p	OR (95% CI)	p	OR (95% CI)	p
	
Asthma	903	1.75 (1.11, 2.76)	0.017	2.76 (1.54, 4.96)	0.001	3.22 (1.62, 6.40)	0.001	1.10 (0.71, 1.71)	0.67
Wheeze	897	1.65 (1.10, 2.46)	0.015	2.84 (1.59, 5.06)	<0.001	3.53 (1.74, 7.18)	<0.001	1.25 (0.87, 1.79)	0.23
Cough	903	2.02 (1.23, 3.33)	0.005	3.48 (1.88, 6.45)	<0.001	4.27 (2.09, 8.72)	<0.001	0.83 (0.49, 1.40)	0.49

All analyses are by logistic regression using the respiratory outcomes as the dependent variables and are adjusted for sex and body mass index. OR = odds ratio, 95% CI = 95% confidence intervals.

There were significant interactions between sex and acid regurgitation symptoms for the bronchodilator response and the FEV_1_/FVC ratio and these findings are shown separately for men and women in table 3. In women, but not men, there were significant associations between reflux symptoms and a lower FEV_1_/FVC ratio and increased bronchodilator responsiveness to salbutamol.

**Table 3:** Associations between lung function, bronchodilator responsiveness and reflux symptoms and irritable bowel syndrome in women and men.

			Heartburn		Regurgitation		Heartburn and regurgitation		Irritable Bowel Syndrome	
		n	Coeff (95% CI)	p	Coeff (95% CI)	p	Coeff (95% CI)	P	Coeff (95% CI)	p
		
FEV_1_/FVC	Women	402	-0.52 (-2.72, 1.68)	0.643	-5.14 (-8.09, -2.20)	0.001	-5.55 (-9.07, -2.04)	0.002	0.04 (-1.58, 1.65)	0.966
	Men	464	0.07 (-1.80, 1.94)	0.942	-0.10 (-2.80, 2.61)	0.944	-0.28 (-3.50, 2.95)	0.866	-0.70 (-2.61, 1.21)	0.473
	p-itn			0.609		0.013		0.029		0.618
										
		n	OR (95% CI)	p	OR (95% CI)	p	OR (95% CI)	P	OR (95% CI)	p
		
BDR	Women	398	3.53 (1.28, 9.70)	0.015	8.74 (2.99, 25.6)	<0.001	11.5 (3.40, 38.6)	<0.001	0.76 (0.25, 2.30)	0.628
	Men	455	1.67 (0.76, 3.68)	0.199	1.11 (0.32, 3.85)	0.864	1.72 (0.48, 6.13)	0.403	1.59 (0.70, 3.61)	0.270
	p-itn			0.256		0.014		0.035		0.300

The FEV_1_/FVC ratio is analysed by linear regression and bronchodilator response by logistic regression. Analyses use these as the dependent variables and are adjusted for BMI. Coeff = regression coefficient, OR = odds ratio, 95% CI = 95% confidence intervals, p-itn = p value for interaction between sex and reflux symptoms, BDR = 10% or greater increase in FEV_1 _following salbutamol.

Atopy (assessed at age 21) was not associated with either heartburn (OR = 0.91, 95% CI: 0.59–1.40, p = 0.67) or regurgitation (OR = 1.04, 95% CI: 0.57–1.90, p = 0.90). The associations between reflux symptoms and asthma diagnosis were not significantly different between those who were atopic and those who were not. However, there were trends to stronger associations between reflux symptoms and wheeze, waking with cough, and bronchodilator responsiveness in those who were not atopic which were of borderline statistical significance (table 4).

**Table 4:** Associations between reflux symptoms and respiratory outcomes in atopic and non-atopic Study members.

	Heartburn			Regurgitation			Heartburn and Regurgitation		
	Non-atopic	Atopic		Non-atopic	Atopic		Non-atopic	Atopic	
	
	OR (95% CI)	OR (95% CI)	p-itn	OR (95% CI)	OR (95% CI)	p-itn	OR (95% CI)	OR (95% CI)	p-itn
	
Asthma	1.81 (0.64, 5.17)	1.96 (1.12, 3.44)	0.873	4.03 (1.11, 14.5)	2.54 (1.23, 5.24)	0.603	3.51 (0.80, 15.3)	3.16 (1.35, 7.38)	0.827
Wheeze	2.88 (1.40, 5.90)	1.30 (0.76, 2.20)	0.072	7.27 (2.23, 23.7)	1.93 (0.95, 3.93)	0.062	6.56 (1.68, 25.6)	2.54 (1.07, 6.00)	0.253
Cough	4.67 (1.98, 11.0)	1.46 (0.73, 2.92)	0.039	5.90 (1.94, 18.0)	2.88 (1.30, 6.38)	0.315	10.8 (3.02, 38.6)	2.87 (1.12, 7.34)	0.099
BDR	2.81 (0.70, 11.3)	2.08 (0.99, 4.36)	0.760	7.57 (1.76, 32.5)	1.76 (0.64, 4.86)	0.108	12.7 (2.71, 59.6)	2.08 (0.67, 6.50)	0.067
									
	Coeff (95% CI)	Coeff (95% CI)		Coeff (95% CI)	Coeff (95% CI)		Coeff (95% CI)	Coeff (95% CI)	
	
FEV_1_/FVC	0.80 (-1.24, 2.84)	-1.15 (-3.13, -0.82)	0.213	-2.43 (-5.37, 0.51)	-2.27 (-4.95, 0.41)	0.940	-1.91 (-5.34, -1.51)	-2.56 (-5.79, 0.66)	0.808

Non-atopic groups (n = 278) and atopic (n = 536) defined by skin-prick tests at age 21. The FEV_1_/FVC ratio is analysed by linear regression. All other analyses use logistic regression. Analyses use the respiratory outcomes as the dependent variables and are adjusted for BMI and sex. OR = odds ratio, Coeff = coefficient, 95% CI = 95% confidence intervals, p-itn = p value for interaction between atopic status and reflux symptoms, BDR = 10% or greater increase in FEV_1 _following salbutamol.

Current smoking was associated with both heartburn (OR = 1.55, 95% CI: 1.04–2.31, p = 0.03) and regurgitation (OR = 1.77, 95% CI: 1.02–3.08, p = 0.04). Adjusting for current smoking or cumulative lifetime pack-year smoking history in addition to sex and BMI did not materially alter any of the analyses.

### Longitudinal associations

**Figure 1:**
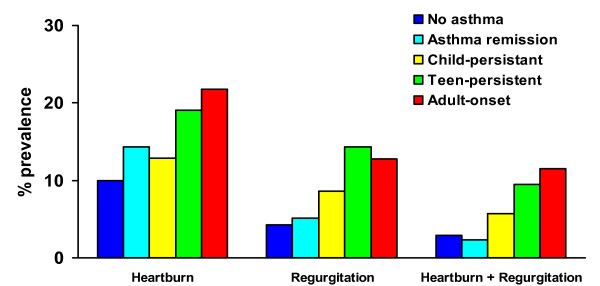
Prevalence of reflux symptoms at age 26 according to history of asthma. No asthma = denies ever having had asthma by age 26 (n = 543). Child-persistent 9 = asthma reported at age 9 or 11 and also at age 26 (n = 70). Teen-persistent = asthma first reported at age 13, 15 or 18 and still present at age 26 (n = 42). Adult-onset = asthma first reported at age 21 or 26 (n = 78). Asthma remission = asthma reported at an earlier age, but not at 26 (n = 174). * = p < 0.05 compared to no asthma.

**Figure 2:**
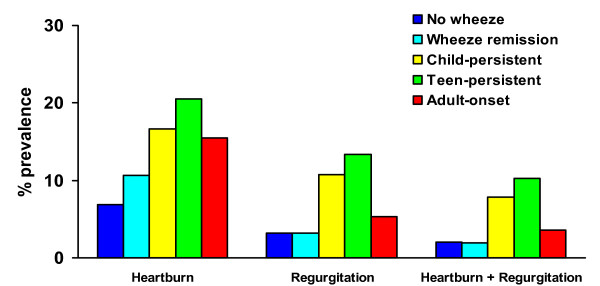
Prevalence of reflux symptoms at age 26 according to history of wheeze. No wheeze = denies ever having had wheeze by age 26 (n = 249). Child-persistent = wheeze at age 9 or 11 and also at age 26 (n = 102). Teen- persistent = wheeze first reported at age 13, 15 or 18 and still present at age 26 (n = 127). Adult-onset = wheeze first reported at age 21 or 26 (n = 168). Wheeze remission = wheeze reported at an earlier age, but not at 26 (n = 174). * = p < 0.05 compared to no wheeze.

Reflux symptoms were not significantly more common in those with persistent asthma since childhood, but were significantly increased in those with asthma since their teens and those with adult onset-asthma compared to those who denied ever having asthma (figure 1). Those with a history of asthma which had remitted by age 26 did not have an increased risk of reflux symptoms. By contrast both childhood-persistent and teen-persistent wheeze were significantly associated with adult reflux symptoms (figure 2). Adult-onset wheeze was significantly associated with heartburn symptoms only. Those who had a history of wheeze which had improved by age 26 did not have a significantly increased risk of reflux symptoms.

Airway hyperresponsiveness to methacholine (or bronchodilator responsiveness in those unable to inhale methacholine) at age 9 years was not significantly associated with reflux symptoms at age 26. However, hyperresponsiveness to methacholine at all subsequent ages was significantly and strongly associated with combined heartburn and acid regurgitation symptoms at age 26 (table 5). These associations were independent of a current asthma diagnosis or current wheeze symptoms at the age at which the methacholine challenge was undertaken (data not shown). There was no association between responsiveness to salbutamol at age 18 and reflux symptoms at age 26. The findings were similar if these analyses excluded the Study members who had had responsiveness to salbutamol measured instead of methacholine challenges at ages 9, 11, 13, 15 and 21 for safety reasons.

**Table 5:** Prediction of reflux symptoms at age 26 years by history of airway hyperresponsiveness.

				Heartburn		Regurgitation		Heartburn and regurgitation	
Challenge agent	Age	n	% with AHR	OR (95% CI)	p	OR (95% CI)	p	OR (95% CI)	p
		
Methacholine*	9	716	16.9	1.40 (0.81, 2.43)	0.234	1.22 (0.55, 2.73)	0.623	1.51 (0.63, 3.61)	0.354
	11	677	10.8	1.62 (0.84, 3.11)	0.150	1.89 (0.80, 4.48)	0.147	3.00 (1.22, 7.40)	0.017
	13	637	8.3	1.63 (0.76, 3.51)	0.210	1.78 (0.66, 4.82)	0.253	2.92 (1.04, 8.17)	0.041
	15	743	8.3	1.92 (1.00, 3.70)	0.051	2.94 (1.35, 6.42)	0.007	3.86 (1.66, 8.97)	0.002
	21	795	7.7	2.59 (1.38, 4.85)	0.003	4.61 (2.26, 9.40)	<0.001	5.56 (2.53, 12.2)	<0.001
Salbutamol	18	758	7.8	1.01 (0.46, 2.21)	0.978	1.13 (0.39, 3.29)	0.818	1.14 (0.34, 3.85)	0.836

At age 18 years, responsiveness to salbutamol bronchodilator was measured. At ages 9, 11, 13, 15 and 21 years responsiveness to methacholine was measured unless a low FEV_1 _precluded methacholine challenge. Airway hyperresponsiveness (AHR) was defined as a PC_20 _methacholine of 8 mg/mL or less or an increase in FEV_1 _of 10% or bronchodilator. OR = odds ratio, 95% CI = 95% confidence intervals. Analyses are by logistic regression using reflux symptoms as the dependent variables and are adjusted for sex.

## Discussion

This study confirms that there is a strong association between symptoms of gastro-oesophageal reflux and symptoms of asthma in this population-based cohort of young adults. These associations were independent of BMI and smoking. Acid regurgitation tended to be a stronger predictor of respiratory symptoms than heartburn, but those with both heartburn and acid regurgitation had the highest risk of respiratory symptoms. The association of reflux symptoms with objective indicators of respiratory function was different for men and women. In women both bronchodilator responsiveness and a lower FEV_1_/FVC ratio were associated with reflux symptoms, whereas in men there was little evidence of an association. The reasons for these sex differences are unclear.

Although this study provides longitudinal follow-up of asthma, wheeze and airway responsiveness since childhood, data on gastro-oesophageal reflux symptoms were not collected during childhood or adolescence and we are unable to establish the temporal sequence between respiratory symptoms and airway responsiveness and gastro-oesophageal reflux. However, symptomatic gastro-oesophageal reflux is uncommon in children and adolescents after infancy [25]. We hypothesised that if asthma precipitates gastro-oesophageal reflux, there would be strong associations between childhood persistent asthma and reflux symptoms. Although childhood wheeze (but not asthma) did significantly predict adult reflux symptoms (figures 1 and 2), teenage-onset asthma and wheeze were better predictors of adult reflux symptoms suggesting that the association between airway and oesophageal dysfunction emerges or strengthens during adolescence. This is supported by the association between airway hyperresponsiveness to methacholine from age 11 onwards and adult reflux symptoms. The strongest association between diagnosed asthma and reflux symptoms was in those with adult-onset asthma, but the findings for wheeze and airway responsiveness are consistent with the hypothesis that longstanding wheeze contributes to the development of reflux, even though it may not have been diagnosed as "asthma".

Perhaps the most striking finding was that airway hyperresponsiveness to methacholine at age 11 years and older predicted the combination of heartburn and acid regurgitation symptoms 15 years later (table 5). These associations were generally similar in males and females (data not shown). By contrast, there was no association between bronchodilator responsiveness at age 18 and adult reflux, while the cross-sectional association between bronchodilator responsiveness and reflux at age 26 was significant in women only. The association between methacholine responsiveness at age 9 and adult reflux was not statistically significant. Methacholine responsiveness was more common at this age and often asymptomatic. For many, this was a self-limiting phenomenon, and long-term associations would not be expected.

Why methacholine responsiveness in later childhood and adolescence predicts gastro-oesophageal reflux symptoms years later is unknown. Episodes of airway narrowing may lead to increased pressure swings in the thorax during the respiratory cycle and promote failure of the gastro-oesophageal sphincter [26]. However, the association was independent of both diagnosed asthma and wheezing symptoms, which suggests that frequent episodes of bronchoconstriction were an unlikely cause of the association. Alternatively, the two phenomena may be linked by altered vagal function, since the vagus nerve controls lower oesophageal tone as well as airway calibre and responsiveness. Autonomic function tests in patients with both asthma and gastro-oesophageal reflux have demonstrated heightened vagal tone, but it is unclear if this was a primary abnormality or a consequence of either asthma, gastro-oesophageal reflux or their treatment [27].

It is possible that the long-term association between teenage methacholine responsiveness and adult reflux symptoms is due to persistence of gastro-oesophageal reflux since adolescence. Although gastro-oesophageal reflux is thought to be uncommon in children and adolescents [25], this may be because it is poorly recognised. In a recent cross-sectional survey, 6% of 13 and 14 year-olds reported having either heartburn or regurgitation symptoms at least once a week in the previous month [28]. Consistent with our findings, reflux symptoms were much more common in the children with asthma. Moreover gastro-oesophageal reflux is often asymptomatic and even "silent" reflux is associated with asthma [29,30]. Several studies have reported improvements in asthma symptoms or lung function after medical or surgical treatment for gastro-oesophageal reflux. Although a recent systematic review found that the evidence was inconsistent and concluded that there was no overall benefit, sub-groups of patients may benefit [31].

The finding that the association between wheeze, waking with a cough, and bronchodilator responsiveness and reflux symptoms tended to be stronger in those who were not atopic would support a hypothesis that gastro-oesophageal reflux causes these symptoms by a mechanism which is distinct from the classic atopic/immunological model of asthma.

The associations between reflux symptoms and asthma were independent of BMI, confirming the finding from the European Community Respiratory Health Survey [13]. This is an important observation since gastro-oesophageal reflux has been suggested as a plausible mechanism for the association between asthma and obesity, particularly since the associations between obesity and both asthma and reflux are stronger in women and because oestrogen has been implicated in both [10,11]. We have previously identified an association between asthma and BMI in women in this cohort [9]. This association between asthma and BMI was not materially altered by including reflux symptoms in the model indicating that reflux does not mediate the asthma-obesity association (data not shown). Perhaps this is not surprising since reflux symptoms in this young adult cohort were only weakly associated with obesity [32].

We found little evidence of an association between asthma and irritable bowel syndrome, which suggests that the association between asthma and gastro-intestinal symptoms is specific for gastro-oesophageal reflux and not a more generalised functional gastro-intestinal disorder. This finding contrasts with results from the postal survey by Kennedy *et al *which found that symptoms of bronchial hyperresponsiveness, irritable bowel syndrome and gastro-oesophageal reflux were all significantly and independently associated with each other [12]. The survey by Kennedy *et al *used a randomly selected sample of adults with a mean age of 38, 12 years older than the participants of this cohort. Moreover, they did not measure lung function but used symptoms to predict bronchial responsiveness.

Strengths of this study include a high rate of follow-up in a population based cohort, prospectively collected data on asthma since childhood, measurements of lung function and airway responsiveness, and directly measured rather than self-reported height and weight. Our findings are coherent across a range of indicators of asthma including a reported diagnosis, wheezing symptoms, and methacholine responsiveness, as well as the symptom of nocturnal cough which could be caused by either asthma or gastro-oesophageal reflux. For women there is also coherence with spirometry and salbutamol-responsiveness. Weaknesses of this study include the fact that detailed information on reflux symptoms was only collected at age 26, and that a subjective measure of "bothersome" symptoms was used to indicate clinically significant reflux. Hence we do not know when these symptoms first occurred, nor do we have data on symptom frequency. However, these factors would reduce the likelihood of identifying significant associations and therefore it is unlikely that these limitations have biased our findings.

## Conclusion

Heartburn and acid regurgitation symptoms are associated with asthma diagnosis, wheeze, and morning cough in this population-based birth cohort followed to age 26. In women, reflux symptoms are also associated with bronchodilator responsiveness and airflow obstruction. The associations were independent of BMI and smoking and tended to be stronger in non-atopic individuals. Early-onset persistent wheeze and airway hyperresponsiveness were associated with adult reflux symptoms. The mechanism of the association warrants further investigation.

## Abbreviations

BDR Bronchodilator response

BMI Body Mass Index

95% CI 95% confidence intervals for mean

FEV_1 _Forced Expiratory Volume in one second

FVC Forced Vital Capacity

OR Odds Ratio

PC_20 _Provoking Concentration to induce a 20% fall in FEV_1_

## Competing interests

Nicholas J. Talley has consulted for AstraZeneca, Axcan, EBMed, Giaconda, Medscape, Solvay, Theravance and Yamanouchi, has received research support from Tap Pharmaceuticals, Novartis, Forest and Merck, and has also received funds for speaking at symposiums from AstraZeneca, TAP, Takeda, ARYx. He does not have a financial relationship with a commercial entity that has an interest in the subject of this manuscript. All other authors: none declared.

## Authors' contributions

RJH analysed the data and drafted the manuscript, RP obtained funding, collected data, provided oversight to the study and critically reviewed the manuscript, DRT provided oversight to the study and critically reviewed the manuscript, JMG analysed data and critically reviewed the manuscript, CRM collected data and critically reviewed the manuscript, JOC collected data and critically reviewed the manuscript, EMF collected data and critically reviewed the manuscript, GPH analysed data and critically reviewed the manuscript, MRS obtained funding, collected data, designed the respiratory section of study and critically reviewed the manuscript, NJT obtained funding, collected data, designed the gastrointestinal section of the study and critically reviewed the manuscript.
